# Terminus-Associated Non-coding RNAs: Trash or Treasure?

**DOI:** 10.3389/fgene.2020.552444

**Published:** 2020-09-15

**Authors:** Wen-Juan Ni, Fuhua Xie, Xiao-Min Leng

**Affiliations:** School of Basic Medicine, Gannan Medical University, Ganzhou, China

**Keywords:** 3′ termini, 3′ UTR, ncRNA, biogenesis, function

## Abstract

3′ untranslated regions (3′ UTRs) of protein-coding genes are well known for their important roles in determining the fate of mRNAs in diverse processes, including trafficking, stabilization, translation, and RNA–protein interactions. However, non-coding RNAs (ncRNAs) scattered around 3′ termini of the protein-coding genes, here referred to as terminus-associated non-coding RNAs (TANRs), have not attracted wide attention in RNA research. Indeed, whether TANRs are transcriptional noise, degraded mRNA products, alternative 3′ UTRs, or functional molecules has remained unclear for a long time. As a new category of ncRNAs, TANRs are widespread, abundant, and conserved in diverse eukaryotes. The biogenesis of TANRs mainly follows the same promoter model, the RNA-dependent RNA polymerase activity-dependent model, or the independent promoter model. Functional studies of TANRs suggested that they are significantly involved in the versatile regulation of gene expression. For instance, at the transcriptional level, they can lead to transcriptional interference, induce the formation of gene loops, and participate in transcriptional termination. Furthermore, at the posttranscriptional level, they can act as microRNA sponges, and guide cleavage or modification of target RNAs. Here, we review current knowledge of the potential role of TANRs in the modulation of gene expression. In this review, we comprehensively summarize the current state of knowledge about TANRs, and discuss TANR nomenclature, relation to ncRNAs, cross-talk biogenesis pathways and potential functions. We further outline directions of future studies of TANRs, to promote investigations of this emerging and enigmatic category of RNA.

## Introduction

The encyclopedia of DNA elements (ENCODE) project aims to reveal functional elements of the human genome, thereby providing new insights into gene and genome functions (Ding et al., 2014; Moraes and Goes, 2016). For instance, RNA sequencing revealed that eukaryotic genomes are pervasively transcribed, using different regions to generate abundant and versatile non-coding RNAs (ncRNAs) (Kapranov et al., 2007; Forrest and Carninci, 2009; Jacquier, 2009; Clark et al., 2011; Jensen et al., 2013; Lu and Lin, 2019). Well-characterized ncRNAs, such as long non-coding RNAs (lncRNAs), small nucleolar RNAs (snoRNAs), and microRNAs (miRNAs), have been found to be variably produced. LncRNAs mainly derive from intergenic regions, introns, and antisense strands (Ayupe et al., 2015; Liu et al., 2015). SnoRNAs mainly arise from introns and intergenic regions. The possible origins of miRNAs resemble those of snoRNAs. Consistently, similar percentages of intronic snoRNAs and intronic miRNAs have been reported in different eukaryotes (Mattick, 2003; Brown et al., 2008; Scott and Ono, 2011). Additionally, many new ncRNAs located at the 3′ and 5′ termini of genes have also been detected (Kapranov et al., 2007; Jacquier, 2009; Djebali et al., 2012; Ma et al., 2017; Laudadio et al., 2018). Owing to the absence of specific patterns in most 3′ end-associated ncRNAs and the limitations of the RNA sequencing technologies, these ncRNAs have usually been ignored for the past decade.

Investigation of the full landscape of 3′ untranslated regions (3′ UTRs) across species and cell types has contributed substantially to our understanding of their biogenesis and functions. Studies on the functions of 3′ UTRs focused primarily on their role in the regulation of gene expression, including mRNA trafficking, translational control, metabolism, and mRNA-protein structures (Wickens et al., 1997; Andreassi and Riccio, 2009; Denti et al., 2013; Jia et al., 2013; Pánek et al., 2016; Mayr, 2017). However, ncRNAs found around 3′ termini are usually not identified as biologically important. Indeed, the presence of terminus-associated small RNAs (TASRs) in both human and mouse genomes was firstly reported in 2007. These RNAs are usually scattered at both strands of protein-coding genes and do not exhibit unique lengths, specific base compositions, or typical secondary structures (Kapranov et al., 2007). Other small RNAs have also been detected at the 3′ ends of genes in both human and chicken genomes (Taft et al., 2009; Wei et al., 2011). Interestingly, these 3′ end-associated small RNAs are significantly different from the characteristic transcription initiation RNAs (tiRNAs) (Taft et al., 2009). In addition, transient transcriptome sequencing (TT-seq) has detected short-lived RNAs downstream of the polyadenylation [poly(A)] sites in human K562 cells. However, these RNAs are difficult to detect as they are usually cleaved from these sites, resulting in unprotected 5′ ends (Schwalb et al., 2016). Thus, these terminus-associated non-coding RNAs (TANRs) did not attract attention due to the lacking of unique length ranges and typical secondary structures.

From a technical perspective, transcriptome sequencing and microarrays show limitations for the discovery of TANRs. Indeed, transcriptome sequencing requires the construction of cDNA libraries and TANRs are often discarded during the rRNA removal step of this process or mixed with annotated transcript fragments afterward. In fact, as mixed fragments, they can partially or completely overlap with the annotated transcripts. Overlapping RNAs can be mapped as part of the annotated transcripts, alternative UTR regions, or even discarded. Furthermore, if some TANRs do not overlap with annotated transcripts, these would be filtered out as erroneous transcripts during bioinformatic analyses. In similarity, transcriptome microarrays are based on available information on annotated transcripts, usually excluding TANRs. Hence, TANRs have been mostly ignored in gene expression studies, given their lack of specific patterns, the uncertainty of their transcriptional origin, and other methodological difficulties (Jacquier, 2009; Yu et al., 2018).

Although TANRs usually are not identified as high-value targets, increasing evidence implied that they are important molecules for several cellular activities. For instance, the detection of diverse TANRs in eukaryotes suggested that they are widespread, abundant, and conserved. Moreover, studies of their biogenesis and functions pointed at TANRs as versatile molecules regulating gene expression. Since the biogenesis and functions of many TANRs are still unclear, and an increasing number of TANRs have been reported, elucidating their biological functions and mechanisms of action has become a new frontier in the field of RNA research.

## Discovery of TANRs

**FIGURE 1:**
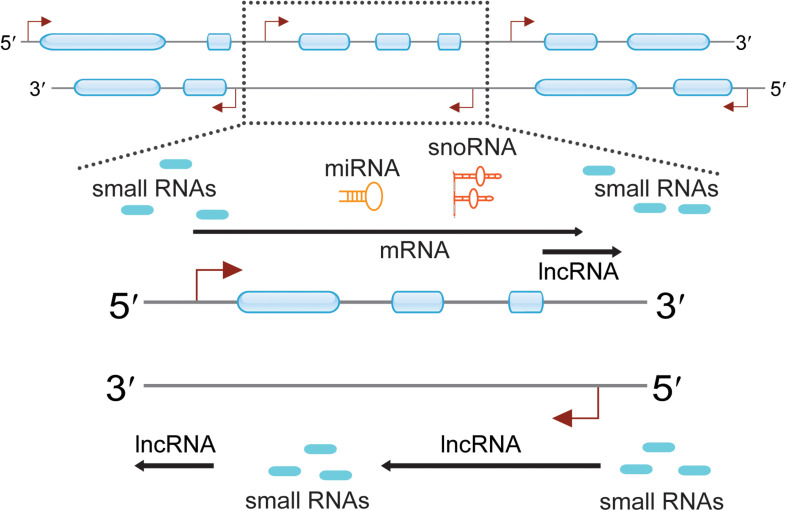
Pervasive transcription across eukaryotic genomes can generate a multitude of diverse RNA species. Genomic regions are indicated with two thin lines marked with direction (5′ to 3′). Exons are presented as blue boxes, and transcription start sites (TTSs) are indicated with red angled arrows. Certain regions of the genome were highlighted and indicated with dotted frame lines. The zoomed in regions enlist many ncRNAs are observed around the protein-coding genes. These ncRNAs include small RNAs, lncRNAs, miRNAs, and snoRNAs.

**FIGURE 2:**
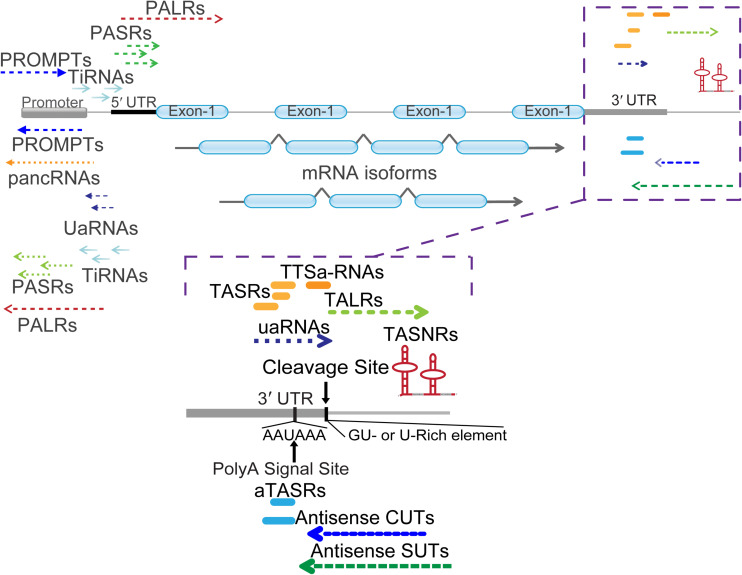
Classification of mRNA 5′ and 3′ end associated ncRNAs. Different types of ncRNAs are indicated with detailed names. PALRs, promoter-associated long RNAs; PROMPTs, promoter upstream transcripts; UaRNAs, upstream antisense RNAs; pancRNAs, promoter-associated non-coding RNAs; PASRs, promoter-associated small RNAs; TiRNAs, transcription-initiation RNAs. The zoomed in regions enlist numerous ncRNAs located at the 3′ termini of mRNA. Cleavage site (AAUAA) and poly(A) signal site (GU- or U-rich element) were indicated with vertical arrow lines and letters. TASRs, terminus-associated small RNAs; aTASRs, antisense TASRs; TASNRs, terminus-associated small nucleolar RNAs; TTSa-RNAs, transcription termination site associated RNAs; TALRs, terminus-associated long RNAs; uaRNAs, 3′ untranslated region (UTR)-associated RNAs; antisense CUTs, antisense cryptic unstable transcripts; antisense SUTs, antisense stable unannotated transcripts.

Applications and breakthroughs of next-generation sequencing (NGS) and gene array in transcriptomics have revealed eukaryotic genomes can generate a multitude of diverse RNA species (Willingham et al., 2006; Kapranov et al., 2007; Jacquier, 2009). Owing to the presence of bidirectional promoters, one more lncRNA and many small ncRNAs have been found around the corresponding mRNA transcription start sites (TSSs) (Seila et al., 2008; Neil et al., 2009; Xu et al., 2009) (Figure 1). These RNAs can be generally termed as promoter-associated RNAs (PARs), including promoter-associated non-coding RNAs (pancRNAs) (Yamamoto et al., 2016; Uesaka et al., 2017), promoter upstream transcripts (PROMPTs) (Preker et al., 2011), upstream antisense RNAs (UaRNAs) (Flynn et al., 2011), stable unannotated transcripts (SUTs), cryptic unstable transcripts (CUTs) (Neil et al., 2009; Xu et al., 2009), promoter-associated long RNAs (PALRs) (Kapranov et al., 2007), tiRNAs (Taft et al., 2009), and other PARs (Jiang et al., 2007). When the attention was shifted to the 3′ terminus, diverse ncRNAs were also discovered. These were divided into different subclasses: TASRs (Kapranov et al., 2007), antisense TASRs (aTASRs) (Kapranov et al., 2010), terminus-associated small nucleolar RNAs (TASNRs) (Leng et al., 2014), transcription termination site associated RNAs (TTSa-RNAs) (Valen et al., 2011; Laudadio et al., 2018), transcription boundary-associated RNAs (TBARs) (Yu et al., 2018), terminus-associated long RNAs (TALRs) (Yue et al., 2010), and 3′ UTR-associated RNAs (uaRNAs) (Mercer et al., 2011) (Figure 2). For a clearer distinction, we highlight that the abbreviation “UaRNAs” stands for “upstream antisense RNAs,” while “uaRNAs” indicates “3′ UTR-associated RNAs.” The different methods used for TANR identification together with the main characteristics of TANRs are summarized in Table 1.

**TABLE 1:** List of TANRs described in this article.

TANRs	Technology	5′ Cap	3′ polyA	Species	Length	Strand	Structure	References
TASRs	Tiling array	No	Yes	*H. sapiens*, *M. musculus* and *A. thaliana*	22–200 nt	Sense	Linear	Kapranov et al. (2007); Djebali et al. (2012), Ma et al. (2017)
aTASRs	Helicos single- molecule sequencing	No	No	*H. sapiens* and *M. musculus*	<200 nt	Antisense	Linear	Kapranov et al. (2010)
TASNRs	Northern blot, RT-PCR, RACE	No	No	*Schizosaccharomyces group*	<200 nt	Sense	Hairpin	Leng et al. (2014)
TTSa-RNAs	RNA sequencing	No	No	*H. sapiens*	22–24 nt	Sense	Linear	Valen et al. (2011); Laudadio et al. (2018)
TALRs	RACE, RT-PCR	Yes	Yes	*H. sapiens*	>200 nt	Sense	Linear	Yue et al. (2010)
uaRNAs	CAGE, SAGE, Microarray	Yes	Yes	*H. sapiens*, *M. musculus* and *D. melanogaster*	>200 nt	Sense	Linear	Mercer et al. (2011)
Antisense CUTs/SUTs	Tiling array, RNA sequencing	Yes	Yes	*S. cerevisiae*	200–500 nt	Antisense	Linear	Neil et al. (2009); Xu et al. (2009)

Terminus-associated small RNAs were firstly reported to cluster at the 3′ termini of mRNAs (Kapranov et al., 2007). In mammals, there are about 200 TASR copies per cell (total numbers for all protein-coding genes), constituting approximately 3% of the small RNA library (Kapranov et al., 2007; Djebali et al., 2012). In *Arabidopsis thaliana*, TASR peaks were identified on 287 protein-coding genes, demonstrating that TASRs mainly accumulated in leaves and young seedlings (Ma et al., 2017). Altogether, the relevance of TASRs in both mammals and plants has been underestimated and the study of these small RNAs did not receive priority in recent years for their lack of specific patterns. Considering their specific location within 3′ UTRs and the presence of poly(A) tails at their 3′ ends, TASRs have been regarded as degraded mRNA products or alternative 3′ UTRs in many studies.

Interestingly, a novel type of TASRs containing polyU tails at their 5′ end have been identified and renamed aTASRs, because they are antisense to 3′ UTRs (Kapranov et al., 2010). There are about 600 aTASR copies per cell, corresponding to 702 RefSeq-annotated protein-coding genes. Meanwhile, 1258 transcripts with non-genomically encoded 5′ poly(U) stretches closely associated with the 3′ termini of known RNAs can also be found in the UCSC Genome Browser database (Kapranov et al., 2010). Since aTASRs display a stretch of U residues at their 5′ ends but no poly(A) at their 3′ ends, they would be discarded in a conventional transcriptome analysis or library construction. Thus, direct RNA sequencing without prior conversion of RNA to cDNA would facilitate the discovery of novel ncRNAs (Furlan et al., 2020).

Argonaute (AGO) proteins are highly specialized binding small RNAs and can regulate gene expression at both transcriptional and posttranscriptional level by interacting with other proteins (Meister, 2013). By sequencing AGO1/2 immunoprecipitated libraries, several TTSa-RNAs were identified in *Homo sapiens*, particularly clustered close to the 3′ termination sites of mRNAs (Valen et al., 2011). Such TTSa-RNAs were found to be originated from 2822 protein-coding genes on average. Additionally, TTSa-RNAs are rich in G residues at their 5′ end and have a peculiar oligo(A) tail at their 3′ end (Laudadio et al., 2018). Compared to TASRs and aTASRs, TTSa-RNAs display shorter lengths (22 to 24 nt) and a specific cellular localization (enriched in nucleus). Beyond linear TASRs, aTASRs, and TTSa-RNAs, hairpin TASNRs (for some given genes) have been found in the yeast species related to *Schizosaccharomyces pombe* Lindner (Leng et al., 2014). In addition to small RNAs, lncRNAs, such as TALRs (for a given gene), uaRNAs (3′ UTR-associated RNAs) (about 1000 copies per cell on average in human), antisense CUTs, and SUTs (about 1000 copies per cell on average), have also been reported (Neil et al., 2009; Xu et al., 2009; Yue et al., 2010; Mercer et al., 2011). Given the evolutionary pressure toward the conservation of 3′ UTR regions, TANRs are usually conserved among different species.

Regarding the genomic location of these ncRNAs, TASRs, aTASRs, TTSa-RNAs, and uaRNAs are located within 3′ UTRs. In particular, TANRs and aTASRs start from poly(A) signal sites, while TTSa-RNAs end at the cleavage sites. Furthermore, TALRs and a small subset of antisense CUTs/SUTs usually overlap with 3′ UTRs. On the other hand, TASNRs are located downstream of 3′ UTRs. As indicated by their name, aTASRs and antisense CUTs/SUTs are located on the antisense strand, while other ncRNAs are located on the sense strand (Figure 2). According to their length (more or less than 200 nt), TALRs, uaRNAs, and antisense CUTs/SUTs are classified as lncRNAs, whereas others are considered small RNAs. Overall, TANRs vary considerably in their genomic location, strand, and length (Table 1).

## Biogenesis of TANRs

Studies of the biogenesis of ncRNAs are required to elucidate their functions and potential roles in the regulation of gene expression (Kim et al., 2009; Li et al., 2010). MiRNAs are currently the best-described small regulatory ncRNAs that follow a specific biogenesis pathway, requiring DROSHA/DGCR8, DICER1, and AGO proteins (Daugaard and Hansen, 2017; Saeed et al., 2020). As TANRs are a novel class of ncRNAs, most but not all proteins associated to their biogenesis are unknown. According to their maturation process, the biogenesis of TANRs can generally occur by one of three models: the same promoter model, the RdRP activity-dependent model, and the independent promoter model.

### The Same Promoter Model

In the same promoter model of TANR biogenesis, firstly, the transcription of TANR precursors is coupled to that of the upstream mRNAs using the same promoter. Then, maturation of TANRs occurs by posttranscriptional cleavage. Considering that the maturation processes of TASRs, TASNRs, TTSa-RNAs, TALRs, and uaRNAs share many characteristics, we summarize them altogether.

Terminus-associated small RNAs are located within the 3′ UTR of genes where no histone modifications marking active promoters or enrichment for RNA polymerase II (RNAPII) occupancy are found (Mercer et al., 2011). Hence, it is reasonable to infer that for their maturation TASRs undergo posttranscriptional cleavage. Studies on the biogenesis of TALRs and TTSa-RNAs also suggested that their maturation mainly depends on posttranscriptional cleavage from the corresponding mRNAs (Yue et al., 2010; Laudadio et al., 2018). However, this maturation process significantly differs from that of miRNAs. Firstly, evidence of the formation of secondary structures and of the corresponding passenger strands, characteristic of miRNA maturation, has not been found for these ncRNAs (Valen et al., 2011). Furthermore, genome-wide studies of TTSa-RNAs also determined that the regions flanking TTSa-RNAs do not tend to form hairpin structures more than randomly picked genomic regions (Laudadio et al., 2018). Secondly, altered expression of DICER and AGO2, required for miRNA biogenesis, had no effects on TTSa-RNA biogenesis (Valen et al., 2011; Laudadio et al., 2018). Importantly, defined sites within the polyA tail and approximately 75% of mRNA 3′ ends carry at least one TTSa-RNA read, suggesting that mRNA 3′ end processing is involved in their biogenesis (Valen et al., 2011). However, TTSa-RNAs are not by-products of mRNA degradation, since they display upstream poly(A) signals and are specifically loaded on AGO proteins. Moreover, TTSa-RNAs tend to carry a G residue in the first position at the 5′ end and an oligo(A) tail (four or more As) at the 3′ end, supporting the hypothesis that TTSa-RNAs undergo posttranscriptional cleavage from the corresponding mRNAs (Laudadio et al., 2018).

Notably, detailed studies on the biogenesis of TASNRs and uaRNAs strongly indicated the same promoter model as the typical one for the biogenesis of most TANRs. In particular, two TASNR precursors (*rpl26*-*snR49* and *rpl29*-*snR93*) highly overlapped with upstream mRNAs; no promoters were detected between mature TASNRs and their precursors; and promoter deletion analysis confirmed that the precursor of TASNR *snR49* and the corresponding upstream mRNA used the same promoter for the regulation of their transcription. Thus, TASNRs undergo processing from precursors during maturation (Leng et al., 2014). As for uaRNAs, no active promoters or enrichment for RNAPII occupancy have been found within the 3′ UTR; however, exon-intron junctions have been detected (Mercer et al., 2011). Moreover, a detailed study on the biogenesis of the uaRNA *FLJ11812* in human cells confirmed that the maturation of this ncRNA depends on posttranscriptional cleavage, and that the TIA1 protein is responsible for this process (Ge et al., 2014).

Although TASNRs and uaRNAs exploit the same promoters of their respective upstream protein-coding genes for transcription, their precursors originate differently. Indeed, uaRNAs may derive from their corresponding mRNAs through maturation by cleavage similarly to TTSa-RNAs. Conversely, TASNR precursors are different transcripts from their corresponding mRNAs, although highly overlapping. As for TASRs and TALRs, it is still unknown whether they are cleaved from their corresponding mRNAs.

### The RdRP Activity-Dependent Model

The RNA-dependent RNA polymerase (RdRP) plays a key role in RNA silencing in fungi, plants, and worms by generating double-stranded RNAs (dsRNAs) from RNA templates (Duempelmann et al., 2020). In the RdRP activity-dependent model of TANR biogenesis, RdRP can *de novo* synthesize antisense TANRs at the 3′ termini of mRNAs by using the sense mRNAs as templates. For instance, it has been reported that the human telomerase reverse transcriptase (TERT) RdRP can perform *de novo* synthesis of short interfering RNAs (siRNAs) that are complementary to template RNAs (Maida et al., 2016). Thus, *de novo* RNA synthesis by RdRP suggests the existence of a novel RNA copying mechanism. Recent studies strongly indicated that the biogenesis of aTASRs depends on RdRP. Indeed, aTASRs contain non-genomically encoded poly(U) stretches at their 5′ ends that are complementary to the 3′ poly(A) tails of mRNAs (Kapranov et al., 2010). These double-stranded and complementary RNAs have been detected in both human cells and plants (Kapranov et al., 2010; Ma et al., 2017). In *A. thaliana*, aTASR fragments were preferentially incorporated into AGO4 and aTASR accumulation was significantly decreased in *rdr2* (RNA-dependent RNA polymerase 2), *nrpd1a* (RNA polymerase IVa), and *nrpd1b* (RNA polymerase IVb) mutants. Thus, RdRPs and RNA polymerase IV are responsible for the biogenesis of some aTASRs, even though the detailed mechanisms remain unknown (Ma et al., 2017). However, the endogenous biochemical pathway that mediates copying of aTASRs in human cells still requires further investigation (Kapranov et al., 2010).

### The Independent Promoter Model

In the independent promoter model, TANRs on the antisense strand have their own promoters. As independent transcripts, their biogenesis is usually regulated by their upstream promoter regions. Although TANRs include only a small number of antisense CUTs/SUTs, several studies indicated that independent promoters are primarily responsible for their biogenesis in *Saccharomyces cerevisiae*. This conclusion derived from the fact that the transcriptional initiation sites of antisense CUTs or SUTs are located in nucleosome-free regions (NFRs), corresponding to promoter regions. Thus, independent transcription is the main biogenesis mechanism of antisense CUTs or SUTs (Neil et al., 2009; Xu et al., 2009).

## Functions of TANRs

**FIGURE 3:**
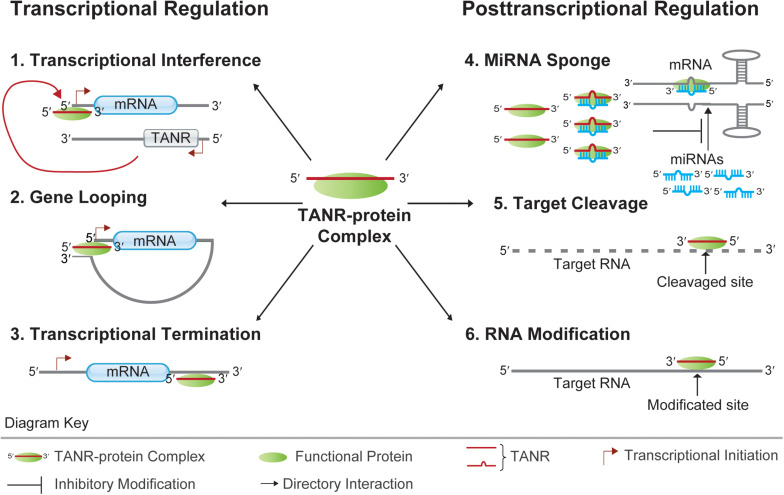
Functions of TANRs. TANRs function at the transcriptional level: 1. Transcriptional Interference: TNAR together with its associated functional protein regulates the transcription of its sense protein-coding gene via binding to the sense promoter region; 2. Gene Looping: TNAR together with its associated functional protein regulates the transcription of its upstream gene by juxtaposing the promoter and terminator together; 3. Transcriptional Termination: TNAR together with its associated functional protein regulates the transcription termination of its upstream gene by binding to the protein-coding gene 3′ UTR. TANRs function at the posttranscriptional level: 4. MiRNA Sponge: TNAR together with its associated functional protein regulates mRNA translation by sponging miRNA from its target mRNA; 5. Target Cleavage: TNAR together with its associated functional protein can direct cleavage of target mRNA at specific site. 6. RNA Modification: TNAR together with its associated functional protein can guide RNA modification at specific site.

The existence of different pathways of TANR biogenesis suggests that they are important for some cellular activities. NcRNAs typically function by forming various ribonucleoproteins (RNPs) together with several proteins. Well-known functional RNP particles include snoRNA ribonucleoproteins (snoRNPs) and miRNA-AGO ribonucleoproteins (miRNPs). These RNPs contain the respective RNAs and a small set of associated proteins (Bachellerie et al., 2002; Bartel, 2004). Within miRNPs, miRNAs usually cause degradation and translational repression of target mRNAs through the formation of miRNA-mRNA duplexes. However, miRNA–mRNA interactions are dynamically regulated by different physiological or pathological conditions (Ni and Leng, 2015). As a group of widely studied functional proteins, AGO proteins associate with a diverse variety of ncRNAs, thereby providing functional and regulatory support for ncRNA-mediated modulation of gene expression (Joshua-Tor and Hannon, 2011; Daugaard and Hansen, 2017). It was reported that TANRs enriched in different subcellular compartments (cytoplasm and nucleus) can interact with different AGO proteins in eukaryotes. Hence, TANRs may regulate gene expression at both transcriptional and posttranscriptional levels (Figure 3).

### Transcriptional Regulation by TANRs

Members of eukaryotic AGO protein family are key players of gene expression (Meister, 2013). Interestingly, a previous study showed that synthetic small RNAs fully complementary to a TALR located beyond the 3′ terminus of progesterone receptor (*PR*) mRNA could modulate *PR* transcription (Younger and Corey, 2011). This provides new insights into the function of TANRs with high nuclear localization. Firstly, the TALR is loaded onto AGO2 upon addition of exogenous miRNA mimics. Then, the complex formed of miRNA mimics, TALR, and AGO2 is recruited to the promoter region of an upstream gene. Finally, a gene loop juxtaposing the promoter and terminator is formed, resulting in altered regulation of transcription (Figure 3) (Yue et al., 2010). Notably, the formation of gene loops is thought to mediate long-distance transcriptional regulation in different eukaryotes (Bratkowski et al., 2018). However, functional studies of AGO1 and AGO2-associated TTSa-RNAs strongly argued against their specific recruitment on chromatin given their nucleoplasm/chromatin abundance, although Gene Ontology (GO) analysis suggested that genes giving rise to TTSa-RNAs are significantly enriched in the regulation of cell cycle progression and DNA integrity checkpoints (Laudadio et al., 2018). On the other hand, evidence of transcriptional stalling via RNAPII backtracking triggering nucleolytic degradation of the nascent RNA indicates that TTSa-RNAs may be implied in the termination of mRNA transcription (Valen et al., 2011). For instance, a recent study in *A. thaliana* indicated that promoter-proximal RNAPII stalling can regulate plant gene transcription (Thomas et al., 2020). Thus, it is reasonable to infer that mammalian TTSa-RNAs might participate in the regulation of gene transcription through the modulation of transcriptional termination (Figure 3).

In plants, AGO1 represses target RNAs in the cytoplasm, while AGO4 usually directs *de novo* DNA methylation in the nucleus (Baulcombe, 2004; Vaucheret, 2008; Carbonell, 2017). Site-specific DNA methylation signals were observed on several genomic loci corresponding to the peaks of many TASRs associated with AGO4 in *A. thaliana* (Ma et al., 2017). Furthermore, some aTASRs are preferentially incorporated into AGO4. Thus, a subset of the TASRs and aTASRs reported in *A. thaliana* may be involved in site-specific DNA methylation (Ma et al., 2017). However, it is not clear if TANR-mediated gene looping is required to guide DNA methylation.

In *S. cerevisiae*, antisense CUTs/SUTs usually couple the transcriptional regulation of neighboring genes. As overlapping and divergent transcripts, they may act as local regulatory signals for transcriptional interference (Figure 3) (Neil et al., 2009; Xu et al., 2009). In addition, transcriptional interference mediated by *cis-*acting antisense CUTs/SUTs involves several chromatin modifiers (such as Set2p, Set1p, Rcoi1p, and Eaf3p) (Nevers et al., 2018). In a recent related report, the transcription of approximately 20% of *S. cerevisiae* genes was found to be repressed by antisense ncRNAs via a chromatin-based transcription interference mechanism. Hence, using near-base-pair-resolution techniques in antisense CUTs/SUTs-inducible strains would reveal the relationship between antisense transcription and repression of sense gene expression, nucleosome occupancy, and transcription-associated histone modifications (Gill et al., 2020).

### Posttranscriptional Regulation by TANRs

Previous reports suggested that 3′ UTRs can function *in trans* to regulate cell proliferation and differentiation in the absence of corresponding protein-coding transcripts (Rastinejad and Blau, 1993; Amack et al., 1999; Jenny et al., 2006). For example, expression of oskar 3′ UTR in *Drosophila* could rescue the egg-less defect of oskar null-mutants in the absence of the Oskar protein. Indeed, the oskar 3′ UTR functions as a scaffold for trafficking and accumulation of Staufen during oogenesis (Jenny et al., 2006). Moreover, in *A. thaliana*, the overexpressed ncRNA *IPS1* can act as a competing endogenous RNA (ceRNA) that positively regulates the expression of *PHO2 by* sequestering *miR-399* from its target site (Franco-Zorrilla et al., 2007). Also, in human embryonic stem cells (hESCs) and human umbilical vein endothelial cells (HUVECs), the uaRNA *FLJ11812* derived from the 3′ UTR of *TGFB2* can be targeted by *miR-4459*. Conversely, uaRNA *FLJ11812* can upregulate the levels of the proteins CDC20B and ATG13, whose coding genes can also be targeted by *miR-4459*. Thus, this uaRNA acts as a ceRNA by sponging *miR-4459* from its target mRNAs (Lu et al., 2015). Therefore, uaRNAs can act as decoys to sponge miRNAs from their target mRNAs (Figure 3). Alternatively, they may act as scaffolds to form regulatory RNA-protein complexes that are functional even in the absence of their corresponding proteins (Mercer et al., 2011).

Structural and functional analyses of ncRNAs in fission yeast suggested that some TANRs act as guide snoRNAs. By forming specific snoRNPs, these snoRNAs can direct methylation or pseudouridylation of target RNAs. Notably, most of such site-specific modifications can affect cell growth *in vivo*. For example, TANR *snR49* was predicted to mediate pseudouridylation of 18S rRNA at the U121 and U305 sites. Upon deletion of TANR *snR49*, the corresponding modifications on rRNA disappeared with consequent delay of cell growth. Furthermore, posttranscriptional modifications of target RNAs by TASNRs are conserved in yeasts (Leng et al., 2014). Thus, TASNRs can act as guide RNAs for targeted RNA modifications (Figure 3).

Since the production of human aTASRs is positively correlated with that of their associated mRNAs, functional studies of aTASRs were based on the corresponding transcripts. These transcripts corresponded to functionally annotated proteins and were further analyzed. Functional enrichment analysis suggested that they are related to translation. Indeed, the GO categories of “structural constituent of ribosome,” “translation,” and “RNA binding” were all significantly overrepresented. Due to the bias of enrichment analysis toward highly synthesized transcripts, all human genes were used as background for a second estimation of enrichment. Nevertheless, similar results were obtained, with the GO biological function category “translation” scoring as the top hit (Kapranov et al., 2010). In *A. thaliana*, aTASRs associated with cytoplasmic AGO1 are proposed to mediate target RNA cleavage (Figure 3) (Ma et al., 2017). Perhaps, synthetic aTASR mimics would help to reveal their mechanism of translational regulation.

## Perspectives and Discussion

Terminus-associated non-coding RNAs were identified years ago, however, their definition is somewhat confused for researchers. Regarding the nomenclature, the abbreviation “UaRNAs” has been used to indicate “upstream antisense RNAs” and sometimes “3′ UTR-associated RNAs.” In terms of timing, upstream antisense RNAs (UaRNAs) were reported before 3′ UTR-associated RNAs (uaRNAs) (Chiu et al., 2006). uaRNAs were then defined according to their specific genomic location within mRNA 3′ UTRs (Mercer et al., 2011). However, UaRNAs were also later discovered and studied (Flynn et al., 2011). Therefore, the abbreviation “uaRNAs” has been given different meanings in separate studies, possibly causing confusion (Chiu et al., 2006; Flynn et al., 2011; Mercer et al., 2011; Lu et al., 2015; Ogami et al., 2017; Yu et al., 2018). To some degree, TTSa-RNAs and TASNRs broadly belong to the same class of TASRs. Indeed, when TASRs were first and systemically described, no identifiable patterns, such as genomic locations, lengths, and subcellular localizations, were unraveled (Kapranov et al., 2007). In contrast, TTSa-RNAs enriched in small RNA libraries of AGO1/2 immunoprecipitates are located before the cleavage sites of mRNAs with restricted lengths (approximately 23 nt) and exhibit nuclear localization (Valen et al., 2011; Laudadio et al., 2018). Moreover, unlike other TANRs, TASNRs are a well-known group of snoRNAs (Leng et al., 2014). Regarding CUTs and SUTs, although no clear partition between CUTs and SUTs exists, some ncRNAs defined as CUTs have been redefined as SUTs (Jacquier, 2009; Neil et al., 2009; Xu et al., 2009). Recently, a uniform annotation system for transcript boundaries has been proposed. This annotation is based on their genomic positions and sequence lengths, and provides suggestions for additional classifications of TANRs, for instance according to their biogenesis pathways, modes of action, and biological outputs (Yu et al., 2018). However, as more and diverse TANRs are found in other eukaryotes, a new, more elaborate nomenclature for TANR classification should be proposed, including detailed information on their genomic location, originating strand, biogenesis pathway, and functions.

The discovery of novel transcripts around annotated transcripts also challenges the concept of gene (Gerstein et al., 2007; Gingeras, 2007). Indeed, not only mRNAs but also lncRNAs can generate functional TANRs. A well-known example is *MALAT1*-associated small cytoplasmic RNA (mascRNA), generated from the nascent lncRNA metastasis associated lung adenocarcinoma transcript 1 (*MALAT1*). MascRNA is located at the 3′ end of mature *MALAT1*, and its maturation is dependent on RNase P (Wilusz et al., 2008). Functional studies of mascRNA found that this ncRNA is involved in cardiovascular innate immunity (Gast et al., 2016). Surprisingly, mascRNA could function as a translational enhancer when placed downstream of *cGFP in vivo* (Wilusz et al., 2012). Studies of the function and biogenesis of mascRNA suggested that TANRs originating from lncRNAs also play an important role in regulating gene expression. Furthermore, several studies have found that ends of both some mRNAs and certain lncRNAs contained conserved secondary structures that might generate TANRs (Kertesz et al., 2010; Nguyen et al., 2016; Yu et al., 2017). Hence, the possible presence of TANRs should not be ignored in either protein-coding or non-protein-coding loci.

Although TANRs can derive from different pathways, their biogenesis might involve the cross-talk of several regulatory mechanisms. For instance, transcription and posttranscriptional processing are important steps of the maturation of TANRs. Furthermore, the carboxy-terminal domain (CTD) of RNAPII is important for coupling mRNA transcription and processing (McCracken et al., 1997; Proudfoot et al., 2002; Ahn et al., 2004; Bentley, 2005). Indeed, by interacting with splicing and 3′ cleavage factors, RNAPII couples transcription, splicing, and cleavage of mRNA precursors (Ahn et al., 2004). Meanwhile, terminal sites are associated with pausing of RNA polymerase (Schwalb et al., 2016). Thus, whether TANRs maturate during a coupled process of transcription and posttranscriptional cleavage or they are derived from RNAPII backtracking remains unknown. Thus, new methods for detecting nascent RNAs or the use of mutants in mRNA 3′ end maturation pathways may shed some light on TANR biogenesis (Wissink et al., 2019; Furlan et al., 2020).

Given the heterogeneity of TANRs, unraveling their functions has become one of the most basic and pressing issues. 3′ UTRs usually harbor critical elements for gene expression, such as miRNA response elements (MREs). Therefore, TANRs that contain MREs may act as miRNA sponges, thus protecting the corresponding mRNAs from translational repression or degradation. For instance, uaRNA *FLJ11812* functions as a ceRNA by sponging *miR-4459* from its target mRNAs, thereby providing a novel direction for functional studies (Ge et al., 2014; Lu et al., 2015). Furthermore, the formation of gene loops juxtaposing the promoter and terminator has been reported in several organisms, and gene looping is thought to mediate long-distance transcriptional regulation (Bratkowski et al., 2018). However, it is unclear whether TANR-mediated gene looping is required for guiding DNA methylation, mRNA processing, or other processes. Nevertheless, the occurrence of miRNA sponging and gene looping provides novel directions for functional studies of sense TANRs. As for antisense ncRNAs, the discovery of chromatin-based transcription interference also suggested a new mechanism of TANR function (Gill et al., 2020).

Once a TANR is discovered, it is challenging to know how to study its function. Basic information, such as the abundance of related mRNAs, the secondary structure, and the subcellular localization of TANRs, aids in understanding their possible functions. For detailed functional studies, induced upregulation and downregulation of TANRs represent an appropriate strategy for primary functional studies. To achieve upregulation, overexpression or synthesis of certain TANRs represents available methods. However, for most TANRs overlapping with certain 3′ UTRs that harbor important regulatory elements, some technical issues need to be overcome to eliminate the potential impact of induced downregulation on the upstream transcripts. Currently, siRNA screens and the application of CRISPR (clustered regularly interspaced short palindromic repeats)-Cas9 to delete certain DNA regions provide useful tools for functional annotation of TANRs in a native context (Zhao et al., 2017).

In summary, the discovery of TANRs in different eukaryotes suggested that they are abundant and conserved. Moreover, studies of the biogenesis and functions of TANRs indicated that they can play important roles in different cellular activities. However, since TANRs represent a novel group of ncRNAs, their biogenesis and functions still require further research. As more information about different TANRs is being reported, their involvement in the regulation of gene expression is due to be unfolded in full and presents one more intriguing observation of the versatility of RNA function.

## Author Contributions

FX and X-ML contributed to the conception of the study. W-JN and X-ML wrote the manuscript. W-JN, FX, and X-ML discussed and improved the revised manuscript. All authors read and approved the final manuscript.

## Conflict of Interest

The authors declare that the research was conducted in the absence of any commercial or financial relationships that could be construed as a potential conflict of interest.
